# Palatal microbiota associated with membranous substances in older Japanese individuals undergoing tube feeding in long-term care: A cross-sectional study

**DOI:** 10.1016/j.heliyon.2023.e20401

**Published:** 2023-09-23

**Authors:** Hironao Asahina, Tadashi Ogasawara, Toshie Akieda, Kohta Miyahara, Yoshiyuki Okada, Kohei Matsumura, Makoto Taniguchi, Akihiro Yoshida, Yasuaki Kakinoki

**Affiliations:** a Department of Oral Health Promotion, Graduate School of Oral Medicine, Matsumoto Dental University, Nagano, 399-0704, Japan; b Matsumoto Dental University, Nagano, 399-0704, Japan; c Yokosuna Dental Clinic, Shizuoka, 424-0035, Japan; d Akieda Dental Clinic, Yamaguchi, 752-0912, Japan; e Department of Special Care Dentistry, Hiroshima University Hospital, Hiroshima, 734-8551, Japan; f Matsumura Dental Clinic, Osaka, 581-0875, Japan; g Oral Microbiota Center, Kagawa, 760-0054, Japan; h Department of Oral Microbiology, Matsumoto Dental University, Nagano, 399-0704, Japan; i Department of Special Needs and Geriatric Dentistry, Kyushu Dental University, Fukuoka, 803-8580, Japan

**Keywords:** Xerostomia, Oral hygiene, Dental care for aged, Microbiota, Tube feeding

## Abstract

**Objective:**

Tube feeders are prone to membranous substance formation on the palate, and those with membranous substances have a risk of fever, with the probable involvement of their oral bacteria. However, the palatal microbiota of those with membranous substances has not been elucidated. Therefore, we evaluated the differences in palatal microbiota between tube-fed individuals with and without membranous substances to clarify the microbiota.

**Materials and methods:**

This study included 19 participants aged 65 years who required tube feeding. The participants’ characteristics were collected from nursing records and oral examinations. If membranous materials were found on the palate, a specimen was collected. Membranous substances were defined as keratotic degeneration observed under a microscope. Additionally, we performed a comprehensive microbiome analysis by extracting DNA from the samples and performing 16 S rRNA gene sequencing. Finally, we compared the participant demographics and oral microbiota between patients with and without membranous substances.

**Results:**

A total of 11 participants had membranous substances associated with “mouth dryness” (p < 0.001) and “constant mouth opening” (p = 0.020). Palatal microbiota differed between those with and without membranous substances. Among the bacteria with a relative abundance greater than 1.0%, the abundance of *Streptococcus* (p = 0.007), *Fusobacterium* (p = 0.041), *Streptococcus agalactiae* (p = 0.009), and *Fusobacterium nucleatum* subsp. *vincentii* (p = 0.026) was significantly higher in the membranous substance group than in the non-membranous substance group.

**Conclusions:**

The palatal microbiota of individuals undergoing tube feeding differed depending on the presence or absence of membranous substances. Membrane substance formation associated with dry mouth purportedly alters the palatal microbiota. *Streptococcus*, *Fusobacterium*, *S*. *agalactiae*, and *F*. *nucleatum* subsp. *vincentii* were more abundant in the oral microbiota of patients with membranous substances. Thus, preventing this formation may help in controlling the growth of these microbes.

## Highlights

• Tube feeders are prone to membranous substance formation on the palate.
• Unlike phlegm and crusts, membranous substances are related to fever in tube feeders.
• Membranous substances are associated with mouth dryness and constant mouth opening.
• Palatal microbiota differed between those with and those without membranous substances.
• Preventing the formation of membranous substance may control unfavorable microbiota.

## 1 Introduction

Membranous substances are often formed in the oral cavity of tube feeders [1,2], on the tongue dorsum, teeth, buccal mucosa, and pharynx, especially on the palate [[1], [2], [3]]. These occurrences can be influenced by various contributing factors, including parenteral intake, dry mouth, constant mouth opening, and impaired consciousness [[1], [2], [3]]. Membranous substances are keratinous degenerations derived from stratified squamous epithelium and mucin from saliva, with partial accumulation of inflammatory cells and bacteria, and are different from phlegm and crusts [4,5]. Some membranous substances bind firmly to the oral mucosa and may cause bleeding when removed [6], and some may fall into the pharynx or larynx and obstruct the airways, resulting in respiratory depression and asphyxia [[7], [8], [9]]. Additionally, fever occurs significantly more frequently in individuals with membranous substances than in those without [10]. Moreover, these cases of fever related to membranous substances may be correlated to the oral microbiota.

In tube-fed older individuals, the oral microbiota has a significantly higher abundance of *Corynebacterium*, *Peptostreptococcus*, and *Fusobacterium* on the tongue coating [11] and *Streptococcus*, *Rothia*, and *Neisseria* on the palate, tongue dorsum, and pharynx compared with the abundance of those found in orally fed individuals [12]. Therefore, the oral microbiota of tube-fed individuals differs from that of orally fed individuals [11,12]. However, the oral microbiota of tube-fed individuals with membranous substances has not been clarified; moreover, the presence of pathogenic bacteria in the oral microbiota of these individuals has not been identified. A comprehensive analysis of the oral microbiota of tube feeders with membranous substances may help identify the bacteria that cause fever and contribute to reducing the risk of fever. Thus, we investigated the palatal microbiota of tube-fed individuals with and without membranous substances and evaluated the differences in their microbiota.

## 2 Materials and methods

### 2.1 Study participants

This study was carried out between May 2017 and May 2019 at Hospital A in Yamanashi Prefecture, Hospital B in Okinawa Prefecture, and Nursing Homes C and D in Okinawa Prefecture, Japan. The inclusion criteria included individuals who aged 65 years or older, required long-term care, were tube-fed (nasogastric feeding or percutaneous endoscopic gastrostomy tube feeding), had no oral intake, and had at least one degree of xerostomia according to the clinical diagnostic classification [13]. The exclusion criteria were established based on the Japanese Association of Rehabilitation Medicine Guidelines for Safety Management [14,15], with some modifications in heart rate, blood pressure, body temperature, and extra systole to match bedridden individuals. Additionally, those who were administered with antimicrobials within 1 month before the commencement of the study as well as those who had pneumonia, cold symptoms, or strong refusal behavior, were excluded (Table 1). This study was approved by the Matsumoto Dental University Ethics Committee (approval no.: 257). A written informed consent was obtained from all participants through their substitute persons.

**Table 1:** Exclusion criteria.

Characteristics	Criteria
Heart rate	More than 100 bpm
Blood pressure	More than 180/110 mmHg
Blood oxygen (SpO_2_)	Less than 90%
Extra systole	Five times per minute
Body temperature	Over 37.0 °C
Clinical findings	With pneumonia and a cold
	With a cough
	With strong refusal behavior
Antimicrobials	History of use within 1 month

### 2.2 Characteristics of participants

Nurses or care staff had administered oral care to the participants at least once a day. We recorded the participants’ sex, age, degree of being bedridden (the degree of independent living for older disabled people, Japanese Ministry of Health, Labour and Welfare, 1991), and underlying medical conditions from hospitalization and admission records. We then confirmed their level of consciousness (Japan Coma Scale), their ability to communicate (i.e., whether they could follow instructions to open their mouth), and the use of moisturizers by the nurse-in-charge.

### 2.3 Oral examination

All oral examinations and sample collection were performed by an experienced dentist (with more than 30 years of experience who was certified by the Japanese Society of Gerodontology). The oral examination was performed in an environmentally controlled room with a temperature of ∼25 °C and relative humidity of ∼30%. We assessed the Community Periodontal Index (World Health Organization, 4th edition), degree of dry mouth (clinical diagnosis classification), presence or absence of remaining teeth, dental caries, and constant mouth opening with the participant in the supine position. The participants’ mouths were deemed to be always open if they were continually open by at least one fingerbreadth, except when their lips were stimulated, in line with a study by Nozawa [16]. Membranous material found on the palatal mucosa was collected using tweezers. The collected membranous material was soaked in a 10% neutral buffered formalin solution. Dental mirrors, probes, tweezers, LED lights, and cameras were used in all assessments.

### 2.4 Diagnosis of membranous substances

The fixed membrane samples were embedded in paraffin. Afterwards, 4.5-μm-thick tissue sections were subjected to hematoxylin and eosin staining. The tissue sections were immersed in xylene, followed by graded ethanol (100–70%), and were then washed with tap water at room temperature. The sections were then immersed in hematoxylin for 5 min and washed thoroughly using tap water. Afterwards, the sections were treated with eosin for 5 min and dehydrated in graded ethanol (70–100%) and xylene. After staining, slides were mounted on glass coverslips. The specimens were considered to contain membranous substances only if degraded keratin derived from the stratified squamous epithelium was observed microscopically [1,2].

### 2.5 Sample collection and palatal microbiome analysis

Samples were obtained by swabbing the palate 20 times using a Forensic Swab XL swab (Sarstedt AG & Co., Nümbrecht, Germany) dipped in saline solution (Normal Saline Syringe OTSUKA 20 mL, Otsuka Pharmaceutical Factory, Tokushima, Japan). The swab was then placed in a 15-mL tube (VIOLAMO tube centrifuge, AS ONE Corporation, Osaka, Japan) containing DNA preservation solution (DNA/RNA Shield, Zymo Research, Irvine, CA, USA) and was agitated with a stirrer for approximately 1 min. The agitated mixture was then refrigerated.

Bacterial DNA was extracted from the samples using MORA-EXTRACT (Kyokuto Pharmaceutical, Tokyo, Japan), according to the manufacturer's instructions. Subsequently, the V3–V4 regions of the 16s ribosomal DNA were amplified by polymerase chain reaction using primers with an overhang sequence added to Illumina MiSeq:341F (5′-TCGTCGGCAGCGTCAGATGTGTATAAGAGACAGCCTACGGGNGGCWGCAG-3′).

And 806R (5′-GTCTCGTGGGCTCGGAGATGTGTATAAGAGACAGGACTACHVGGGTATCTAATCC-3′) (Takara Bio Inc., Shiga, Japan). The amplified samples were analyzed using a next-generation sequencer (MiSeq; Illumina, San Diego, CA, USA) at the Oral Microbiota Center (Kagawa, Japan). The obtained sequence data were subjected to read processing using Usearch (version January 8, 1861, https://www.drive5.com/usearch/), and sequences shorter than 400 bases were excluded from the analysis [17]. Chimeric sequences were then removed from the dataset using ChimeraSlayer [18]. Operational taxonomic units (OTUs) were sorted using the UCLUST [19] algorithm with a cutoff of 97% base similarity. Each OTU was matched to the Human Oral Microbiome Database (version 14.51; http://www.homd.org) [20] to identify the bacterial species.

Diversity analysis and UniFrac [21] distance calculations were performed on the data with chimeric sequences removed using Quantitative Insights into Microbial Ecology (version 1.9.1, QIIME, http://qiime.org) [17]. To assess beta diversity, we performed Principal Coordinate Analysis (PCoA) using the Weighted UniFrac distance to visualize the microbiota similarity between each sample in a scatter plot. The scores of Shannon's and Simpson's indices were calculated to assess alpha diversity, indicating the evenness and richness of the microbial community in each sample.

### 2.6 Statistical analyses

We used the Fisher's exact and χ^2^ tests to compare patient characteristics and comorbidities in the groups with and without membranous substances. The Shapiro-Wilk normality test was used to assess whether alpha diversity and mean relative abundance were normally distributed. Data from the two membranous substance groups were then compared using the Mann-Whitney *U* test for the Simpson index of alpha diversity and mean relative abundance, whereas the Shannon index of alpha diversity was compared using an unpaired *t*-test after performing to f-test. Permutational multivariate analysis of variance (PERMANOVA), with the Adonis function in the Vegan package conducted in R, using the matrix of weighted UniFrac distance, was performed to compare the differences in bacterial community structure between the groups with and without membranous substances. Correlation ratios were calculated to examine the association between participant characteristics, comorbidities, and palatal microbiota. The Fisher's exact, χ^2^, Shapiro-Wilk normality test, unpaired *t*-test, f-test, and Mann–Whitney U tests were performed using the EZR ver. 1.4 (Jichi Medical University, Saitama, Japan) [22]. Correlation ratios were calculated using the Excel statistical analysis software ver. 8.4 (iStat Corporation, Tokyo, Japan). Statistical significance was set at a p-value of <0.05.

## 3 Results

### 3.1 Participants’ characteristics and comorbidities

Table 2, Table 3 present the participants’ characteristics and comorbidities. All participants in the study had dry mouth, and the formation of membranous substances was observed in participants with severely dry mouths (Class III). A total of 11 patients had membranous substances, which were significantly associated with “constant mouth opening” (p = 0.020) and “mouth dryness” (p < 0.001). No significant associations were identified in terms of comorbidities among the participants.

**Table 2:** Characteristics of participants with membranous substances (n = 19).

Characteristics		Membranous substances		
		*With* (%)	*Without* (%)	p-value
*Sex*				
Male		6 (31.6)	7 (36.8)	0.177^a^
Female		5 (26.3)	1 (5.3)	
*Age group (years)*				
69<		1 (5.3)	0 (0.0)	0.833^b^
70–79		3 (15.7)	4 (21.1)	
80–89		6 (31.6)	3 (15.7)	
≥90		1 (5.3)	1 (5.3)	
*Bedridden level*				
J Rank		0 (0.0)	0 (0.0)	–
A Rank		0 (0.0)	0 (0.0)	
B Rank		0 (0.0)	0 (0.0)	
C Rank		11 (57.9)	8 (42.1)	
*Japan Coma Scale*				
I		4 (21.1)	4 (21.1)	0.658^a^
II		7 (36.7)	4 (21.1)	
III		0 (0.0)	0 (0.0)	
*Communication*				
Able		3 (15.7)	4 (21.1)	0.377^a^
Unable		8 (42.1)	4 (21.1)	
*Location*				
Yamanashi	A	3 (15.8)	0 (0.0)	0.086^b^
Okinawa	B	1 (5.3)	1 (5.3)	
	C	4 (21.1)	7 (36.7)	
	D	3 (15.8)	0 (0.0)	
*Oral moisturizers*				
Used		8 (42.1)	8 (42.1)	0.228^a^
Not used		3 (15.8)	0 (0.0)	
*Remaining teeth*				
Edentulous		2 (10.5)	3 (15.8)	0.603^a^
≥1		9 (47.4)	5 (26.3)	
*Community Periodontal Index*				
Edentulous		2 (10.5)	3 (15.7)	0.944^b^
0		0 (0.0)	0 (0.0)	
1		1 (5.3)	1 (5.3)	
2		4 (21.1)	3 (15.7)	
3		1 (5.3)	0 (0.0)	
4		3 (15.8)	1 (5.3)	
*Caries*				
Edentulous		2 (10.5)	3 (15.8)	0.773^b^
0		1 (5.3)	0 (0.0)	
≥1		8 (42.1)	5 (26.3)	
*Constant mouth opening*				
Yes		8 (42.1)	1 (5.3)	0.020^a^
No		3 (15.8)	7 (36.8)	
*Mouth dryness*				
Class I		0 (0.0)	3 (15.7)	<0.001^b^
Class II		0 (0.0)	4 (21.1)	
Class III		11 (57.9)	1 (5.3)	

Notes: Bedridden level: J Rank: Have some sort of disability, but are almost independent in daily life and can get out of their home without assistance; A Rank: Almost independent for indoor daily life, but cannot go outside without care; B Rank: Require some sort of care for indoor daily life and stay in bed for most of the time but can keep a sitting position; C Rank: Stay all the time in bed and require care for toileting, eating, and changing clothes. Japan Coma Scale I: the patient is awake without any stimuli; II: the patient can be aroused (then reverts to the previous state after stimulation cessation); III: the patient cannot be aroused with any forceful mechanical stimuli. Communication: Able to follow mouth opening instructions; unable to follow mouth opening instructions. Community Periodontal Index: 0: healthy; 1: bleeding observed, directly or by using a mouth mirror, after probing; 2: calculus detected during probing; however, all of the black bands on the probe were visible; 3: Pocket 4–5 mm (gingival margin within the black band on the probe); 4: pocket 6 mm or more (black band on the probe not visible). Constant mouth opening: Yes: Resting mouth opening is always greater than the fingerbreadth; No: Resting mouth always opens less than the fingerbreadth. Mouth dryness: I: Saliva shows viscosity; II: Saliva shows tiny bubbles on the tongue; III: Dry tongue without viscosity, little or no saliva.

a Fisher's exact test.

b *x*^2^, test.

**Table 3:** Comorbidities with membranous substances (n = 19).

Comorbidities	Membranous substances		
	*With* (%)	*Without* (%)	p-value
*Cranial nerve disorders*			
Yes	11 (57.9)	8 (42.1)	–
*Cardiovascular disease*			
Yes	5 (26.3)	3 (15.8)	1
No	6 (31.6)	5 (26.3)	
*Endocrine disease*			
Yes	4 (21.1)	2 (10.5)	1
No	7 (36.8)	6 (31.6)	
*Respiratory disease*			
Yes	3 (15.7)	4 (21.1)	0.377
No	8 (42.1)	4 (21.1)	
*Gastrointestinal disease*			
Yes	2 (10.5)	3 (15.8)	0.603
No	9 (47.4)	5 (26.3)	
*Renal urological disease*			
Yes	3 (15.7)	4 (21.1)	0.377
No	8 (42.1)	4 (21.1)	
*Liver disease*			
Yes	1 (5.3)	1 (5.3)	1
No	10 (52.6)	7 (36.8)	
*Bone and joint disease*			
Yes	2 (10.5)	1 (5.3)	1
No	9 (47.4)	7 (36.8)	

Notes: The Fisher's exact test was used for all comorbidities.

### 3.2 Alpha and beta diversity

**Fig. 1:**
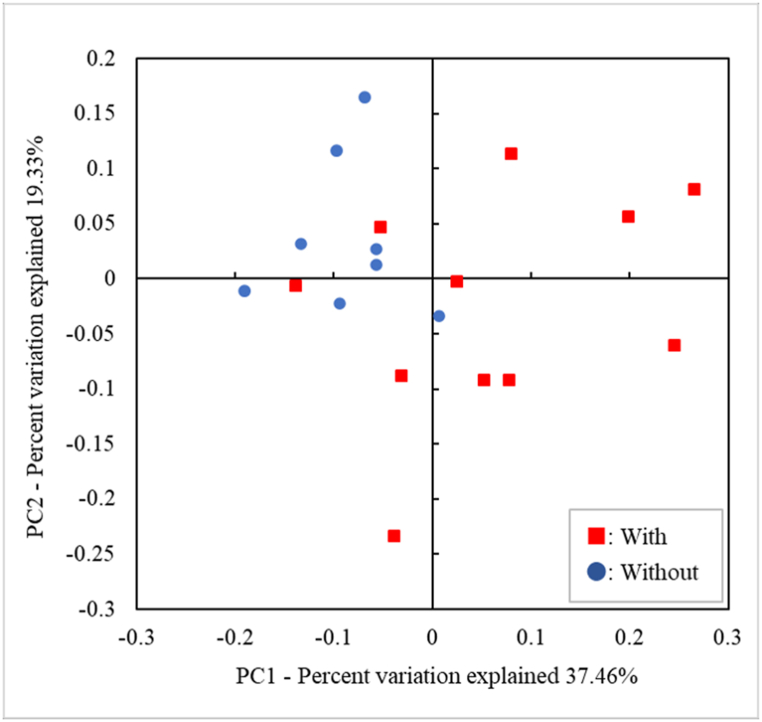
Similarity relations among the palatal microbiota compositions of the 19 participants using a Principal Coordinate Analysis. The plots are generated using weighted UniFrac distances. These two components elucidate a 56.79% variance.

The Shannon and Simpson indices did not show significant differences in alpha diversity (Table 4). To assess the beta diversity and based on the presence or absence of membranous substances, we prepared a PCoA plot based on weighted UniFrac and prepared a scatter plot. Samples with membranous substances were widely distributed, whereas those without membranous substances were concentrated in the second quadrant; this highlighted the different aspects of the two groups in the plot. This difference was statistically significant according to the PERMANOVA (p = 0.0100) (Fig. 1).

**Table 4:** Comparisons using the Shannon and Simpson diversity indices of those with and without membranous substances.

Indices	Membranous substances		
	*With* (median)	*Without* (median)	p-value
Shannon diversity index (Mean ± SD)	3.4 ± 0.9 (3.51)	3.3 ± 0.7 (3.27)	0.936^a^
Simpson diversity index (Mean ± SD)	0.8 ± 0.1 (0.830)	0.8 ± 0.1 (0.815)	0.649^b^

SD, Standard Deviation.

a Unpaired *t*-test.

b Mann–Whitney *U* test.

### 3.3 Palatal microbiota composition

The sequences were assigned to 260 species-level OTUs. In total, there were 112 genera. Seventeen species and 14 genera were identified, with a mean relative abundance of >1.0%. Subsequently, bacteria with a mean relative abundance of >1.0% were compared, demonstrating a higher prevalence of the genera *Streptococcus* (p = 0.009), *Fusobacterium* (p = 0.026), *Streptococcus agalactiae* (p = 0.007), and *Fusobacterium nucleatum* subsp. *vincentii* (p = 0.041) in the membranous substance group than in the group without membranous substances (Table 5, Table 6).

**Table 5:** Relative abundance comparisons between those with and without membranous substances (species level).

Species	Relative abundance (Mean ± SD) (%)			
	Overall	With	Without	p-value
*Neisseria flavescens*	18.2 ± 18.1	24.5 ± 20.3	9.5 ± 8.1	0.075
*Rothia mucilaginosa*	17.3 ± 12.7	12.5 ± 8.4	24.0 ± 15.0	0.075
*Streptococcus* sp. oral taxon 058	10.1 ± 7.7	11.6 ± 8.0	8.0 ± 7.3	0.272
*Streptococcus agalactiae*	8.9 ± 13.6	14.2 ± 15.9	1.5 ± 2.2	0.009
*Neisseria* sp. oral taxon 020	5.9 ± 9.3	1.2 ± 1.7	12.5 ± 11.5	0.126
*Haemophilus haemolyticus*	3.9 ± 7.8	2.3 ± 2.9	6.1 ± 11.6	0.778
*Agrobacterium tumefaciens*	3.4 ± 14.2	0.0 ± 0.1	8.0 ± 21.9	0.121
*Fusobacterium nucleatum* subsp. *vincentii*	2.4 ± 3.5	3.3 ± 3.9	1.2 ± 2.8	0.026
*Aggregatibacter* sp. oral taxon 512	2.4 ± 4.2	3.4 ± 5.1	1.0 ± 2.0	0.316
*Lactobacillus crispatus*	2.0 ± 8.7	3.5 ± 11.5	0.0 ± 0.0	0.452
*Neisseria elongate*	2.0 ± 5.7	2.2 ± 6.3	1.8 ± 5.0	0.103
*Pseudomonas aeruginosa*	1.7 ± 3.9	1.3 ± 3.7	2.3 ± 4.3	0.472
*Moraxella catarrhalis*	1.5 ± 4.6	2.0 ± 5.8	0.8 ± 2.3	0.893
*Neisseria bacilliformis*	1.3 ± 2.9	1.0 ± 2.6	1.8 ± 3.3	0.435
*Neisseria mucosa*	1.2 ± 1.6	1.2 ± 1.5	1.3 ± 1.9	0.901
*Porphyromonas pasteri*	1.1 ± 1.8	1.4 ± 1.5	0.8 ± 2.2	0.075
*Delftia acidovorans*	1.1 ± 2.7	0.1 ± 0.1	2.4 ± 3.9	0.481

Notes*:* Only overall relative abundances of >1.0% are presented. Statistical differences were calculated using the Mann–Whitney *U* test.

SD, Standard Deviation.

**Table 6:** Relative abundance comparisons between those with and without membranous substances (genus level).

Genus	Relative abundance (Mean ± SD) (%)			
	Overall	With	Without	p-value
*Neisseria*	29.3 ± 16.9	30.1 ± 19.3	28.1 ± 14.0	0.840
*Streptococcus*	19.1 ± 13.3	26.0 ± 12.5	9.7 ± 7.2	0.007
*Rothia*	17.4 ± 12.6	12.5 ± 8.4	24.1 ± 14.8	0.075
*Haemophilus*	5.0 ± 8.2	4.1 ± 4.8	6.2 ± 11.6	0.492
*Agrobacterium*	3.4 ± 14.2	0.0 ± 0.1	8.0 ± 21.9	0.121
*Fusobacterium*	2.8 ± 3.5	3.9 ± 3.7	1.4 ± 2.9	0.041
*Aggregatibacter*	2.5 ± 4.1	3.6 ± 5.0	1.0 ± 2.0	0.238
*Porphyromonas*	2.3 ± 2.0	2.3 ± 1.9	2.2 ± 2.2	0.84
*Lactobacillus*	2.0 ± 8.7	3.5 ± 11.5	0.0 ± 0.0	0.086
*Prevotella*	1.8 ± 1.9	1.9 ± 2.2	1.7 ± 1.5	0.968
*Pseudomonas*	1.7 ± 3.9	1.3 ± 3.7	2.3 ± 4.3	0.472
*Moraxella*	1.5 ± 4.6	2.0 ± 5.8	0.8 ± 2.3	0.637
*Actinomyces*	1.4 ± 1.9	0.9 ± 1.3	2.1 ± 2.4	0.109
*Delftia*	1.1 ± 2.7	0.1 ± 0.1	2.4 ± 3.9	0.481

Notes*:* Only overall relative abundances of >1.0% are presented. Statistical differences were calculated using the Mann–Whitney *U* test.

SD, Standard Deviation.

### 3.4 Palatal microbiota correlation with participants’ characteristics

Correlation ratios were calculated between the mean relative abundance of the identified 50 most abundant genera and the participants’ characteristics and comorbidities (Table S1). *Streptococcus* correlated with “membranous substances” (r = 0.390; p = 0.004), “Mouth dryness” (r = 0.336; p = 0.038), and “sex” (r = 0.221; p = 0.043), while *Fusobacterium* correlated with “constant mouth opening” (r = 0.434; p = 0.002).

## 4 Discussion

The oral microbiota of tube-fed and orally fed patients differ [11,12]; however, studies on the oral microbiota of tube-fed patients based on their oral environment are lacking. The oral hygiene status of tube-fed patients is poor [23], which may alter the oral microbiota. In the present study, the palatal microbiota was compared in the presence or absence of membranous substances, which often form in tube-fed patients, and the results showed different microbiota.

Tube feeders requiring nursing care are more prone to dry mouth due to constant mouth opening [1,2], with an odds ratio of 11.2 (95% CI = 0.880–143.27), which is significantly higher compared with in oral feeders [24]; therefore, tube feeders are more likely to have membranous substances in their oral cavity [1,2,5]. In this study, membranous substances were associated with “constant mouth opening” and “mouth dryness,” and severe dry mouth was observed in those who had membranous substances in their oral cavity. Since dry mouth affects oral microbiota [25], severe dry mouth accompanied by the formation of membranous substances may alter the palatal microbiota in tube-fed patients. However, the alpha diversity did not differ between the groups, likely because tube feeders already have low alpha diversity [12].

Moreover, we compared bacteria with relative abundances greater than 1.0% between those with and without membranous substances and reported *Streptococcus*, *Fusobacterium*, *S. agalactiae*, and *F. nucleatum* subsp. *vincentii* in the group with membranous substances. *S. agalactiae* (Group B *Streptococcus*: GBS) are rarely found in the oral cavity of orally fed individuals but are more common in the oral cavity of tube-fed individuals [11,26]. Interestingly, in our study, GBS was significantly higher in the group with membranous substances than in the group without, suggesting that GBS may become more abundant with the formation of membranous substances in tube-fed individuals. The adverse health effects of GBS on tube-fed individuals are concerning. A recent report showed that the incidence of GBS-induced infections in older individuals was increasing [27]. For example, GBS pneumonia almost exclusively occurs in debilitated older individuals [27] and is caused by bacteremia or transtracheal aspiration [28]. Similarly, GBS pneumonia in tube feeders has been extensively reported [29]. Thus, GBS is an important challenge in older individuals with comorbidities [30].

*Streptococcus* found to be associated with “membranous substances,” “mouth dryness,” and “sex.” *Streptococcus* is common in oral cavity with dry mouth [25]; therefore, it is plausible that a similar association was observed in our study. In the present study, the correlation ratio for “membranous substances” was stronger than for “mouth dryness” owing. This could be attributed to the severe dry mouth promoting the formation of membranous substances, thereby further aggravating to the oral environment due to their presence. Regarding the association with “sex,” further research is required to elucidate the underlying relationship.

Dry mouth [31] and tube feeding [32,33] are associated with pneumonia; moreover, *Streptococcus* infection is a risk factor for aspiration pneumonia [34,35]. Furthermore, membranous substances are associated with fever [10]. Therefore, respiratory infections from GBS or *Streptococcus* spp. are suspected to cause fever in individuals with membranous substances.

The possible routes for bacterial infection in the palate are hematogenous infections from the bleeding site [6] caused by the removal of membranous substances and infection due to membranous substances falling into the pharynx, larynx, and trachea [[7], [8], [9]]. Additionally, microbes in the palate, tongue dorsum, and pharynx were similar among tube-fed patients [12]. Therefore, bacteria in the palate can easily migrate to the pharynx and could lead to respiratory infection if aspirated into the trachea.

The genera *Fusobacterium* and *F. nucleatum* sub spp. *Vincentii*, which are associated with periodontal disease [36,37], were identified in high abundance in individuals with intraoral membranous substances. Additionally, *Fusobacterium* was not correlated with “membranous substances,” “mouth dryness,” or “Community Periodontal Index; ” however, it was correlated with “constant mouth opening,” which was associated with the formation of membranous substances. As mentioned in previous reports [25,38], the abundance of these bacteria may increase due to dry mouth. The lack of correlation between *Fusobacterium* and the “Community Periodontal Index” may be owing to the low percentage of participants with gingival bleeding or deep periodontal pockets.

The two bacterial species and two genera that showed significant differences in this study were hypothesized to increase relative to the severity of dry mouth and the subsequent formation of membranous substances. Thus, preventing the formation of membranous substances may help control the abundance of these bacteria.

Cleaning the oral mucosa and applying oral moisturizers are effective methods for avoiding dry mouth and preventing the formation of membranous substances [9,39,40]. However, as the effect of moisturizers is short-lasting [[41], [42], [43]], membranous substances begin to re-form and stick strongly to the oral mucosa 12–24 h after oral care [40]. Therefore, it is important to perform oral care at 6–12 h intervals [40] as it would be easier to remove these membranous substances before they become firmly attached to the oral mucosa. In addition, previous studies have observed that severe dry mouth is associated with the presence of membranous substances firmly attached to the mucosa [1,2,40]. Based on these findings, it is plausible to consider that severe dry mouth in tube-fed patients may have contributed to the increased prevalence of the bacteria observed in this study. The continuous use of moisturizers in individuals with severe dry mouth is thought to promote oral microbiota that is similar to that of individuals without dry mouth [44]. Applying moisturizers may reduce the number of bacterial species and control the proliferation of harmful bacteria. It has been suggested that frequent moisturization and oral mucosal care, including the prevention of membranous substance formation, are essential and may reduce the risk of fever in people who have intraoral membranous substances.

### 4.1 Limitations and perspectives

A limitation of this study was its small sample size. However, the target population was highly limited and recruiting participants was challenging. Future studies are required to validate our results in larger populations.

Because this was a cross-sectional study that focused on identifying the palatal microbiota of participants with membranous substances, it is unknown how membranous substance removal affects the palatal microbiota. Moreover, it is unclear how the microbiota changes with the formation of membranous substances and whether the abundance of the bacteria observed in this study actually increases with their formation. In future studies, we plan to collect bacteria chronologically after oral care and observe how the microbiota changes with oral dryness and the formation of membranous substances. Furthermore, our objective is to conduct interventional studies to investigate the potential benefits of removing membranous substances and providing professional oral care with moisturization. Through these studies, we aim to assess whether such interventions can effectively reduce the incidence of fever and pneumonia in this population. This will enable us to consider the relationship between fever or pneumonia and oral microbiota, which could lead to the stratification of patients. If people who have membranous substances in their oral cavities have fever, a respiratory infection such as pneumonia can be suspected, and prompt interventions are possible. The removal of membranous substances can improve the health of older adults who require nursing care.

## 5 Conclusion

In the present study, the palatal microbiota differed depending on the presence or absence of membranous substances. In addition, significantly more pneumonia-associated bacteria were identified in the group with membranous substances than in the group without membranous substances. Finally, the formation of membranous substance is associated with dry mouth and purportedly alters the palatal microbiota; thus, preventing the formation of these membranous substances may be helpful in controlling unfavorable microorganisms.

## Author contribution statement

Hironao Asahina: Tadashi Ogasawara: Conceived and designed the experiments; Performed the experiments; Analyzed and interpreted the data; Wrote the paper. Toshie Akieda: Kohta Miyahara: Performed the experiments; Analyzed and interpreted the data. Yoshiyuki Okada: Analyzed and interpreted the data; Wrote the paper. Kohei Matsumura: Performed the experiments. Makoto Taniguchi: Contributed reagents, materials, analysis tools or data. Akihiro Yoshida: Contributed reagents, materials, analysis tools or data; Wrote the paper. Yasuaki Kakinoki: Conceived and designed the experiments; Wrote the paper.

## Funding

This work was supported by 10.13039/501100001691JSPS KAKENHI, Grant Number: JP18K09897 (Principal Investigator: Tadashi Ogasawara).

## Data availability statement

Data will be made available on request.

## Declaration of competing interest

The authors declare that they have no known competing financial interests or personal relationships that could have appeared to influence the work reported in this paper.
